# Case Report: staged electron beam radiotherapy for inoperable primary ectopic abdominal extramammary Paget disease

**DOI:** 10.3389/fonc.2026.1923850

**Published:** 2026-08-19

**Authors:** Jiarun Jiang, Chunxu Yang, Qingming Xiang, Dazhen Jiang, Li Niu, Jie Cheng, Xiaojia Chen, Sen Shi, Jun Lin, Hui Qiu, Shaoxing Sun

**Affiliations:** 1 Department of Gastroenterology, Hubei Provincial Clinical Research Center for Intestinal and Colorectal Diseases, Hubei Key Laboratory of Intestinal and Colorectal Diseases, Zhongnan Hospital of Wuhan University, Wuhan, China; 2 Department of Radiation and Medical Oncology, Hubei Key Laboratory of Tumor Biological Behaviors, Hubei Cancer Clinical Study Center, Zhongnan Hospital of Wuhan University, Wuhan, China; 3 Department of Pathology, Zhongnan Hospital of Wuhan University, Wuhan, China; 4 Department of Gastroenterology, Wuhan Fourth Hospital, Wuhan, China

**Keywords:** bolus, case report, ectopic extramammary Paget disease, electron beam radiotherapy, field coverage

## Abstract

Extramammary Paget disease (EMPD) is a rare cutaneous adenocarcinoma that usually arises in apocrine gland-bearing areas, whereas primary ectopic EMPD of the abdominal skin is exceptionally uncommon. We report a 79-year-old woman with a five-year history of progressively enlarging eczema-like lower abdominal-pelvic skin lesions, including erosion, exudation, scaling, crusting, and pruritus, initially treated as chronic dermatitis without improvement. Histopathology showed large atypical Paget cells with focal dermal microinvasion; immunohistochemistry was positive for cytokeratin 7 (CK7) and GATA-binding protein 3 (GATA3) and negative for cytokeratin 20 (CK20) and p40, while Ki-67, a proliferation marker, showed a labeling index of approximately 60%. Because radical surgery was unsuitable, she received response-adapted two-stage electron beam radiotherapy. The first stage delivered 43.2 Gy in 12 every-other-day fractions using 12-MeV electrons, a 5-mm tissue-equivalent bolus, and customized lead shielding, followed by a 10 Gy boost in five daily fractions using 9-MeV electrons for residual visible disease. The treated field showed complete clinical resolution approximately one month after radiotherapy, with no in-field recurrence nearly one year later. Residual erythematous scaly plaques persisted outside the original field. This case supports response-adapted electron beam radiotherapy as a feasible non-surgical option for selected inoperable superficial EMPD and emphasizes the practical importance of pretreatment mapping, adequate field coverage, bolus conformity, shielding, and field-edge planning for extensive lesions on curved body surfaces.

## 1 Introduction

Extramammary Paget disease (EMPD) is an uncommon cutaneous adenocarcinoma with an estimated annual incidence of 0.01-0.24 per 100,000 population. It most often affects apocrine gland-rich sites such as the vulva, perianal region, scrotum, penis, groin, and axilla, and may be intraepidermal or invasive with nodal or distant spread (1, 2). Primary ectopic EMPD arising on abdominal skin is exceptionally rare. Because EMPD can resemble chronic eczema, fungal infection, contact dermatitis, or psoriasis, diagnosis may be delayed, particularly when lesions occur outside classical sites.

Surgery remains the standard curative treatment for localized EMPD, including wide local excision or Mohs micrographic surgery when feasible (3–5). Radiotherapy is an important alternative for patients who are medically inoperable, decline surgery, have unresectable lesions, or require postoperative treatment (6–8). However, the optimal dose, field arrangement, bolus use, shielding, and field-edge strategy are not well defined for extensive superficial EMPD on curved body surfaces. We report a rare case of primary ectopic abdominal EMPD treated with response-adapted two-stage electron beam radiotherapy, highlighting both the favorable in-field response and the challenges of geographic coverage.

## 2 Case description

### 2.1 Clinical history and physical examination

A 79-year-old woman presented with a five-year history of progressively enlarging eczema-like lesions involving the lower abdominal-pelvic skin. The lesions were erythematous, erosive, exudative, scaly, crusted, and intermittently pruritic, without obvious pain. She had previously been managed as having chronic dermatitis with topical corticosteroids, oral antihistamines, and systemic anti-infective treatment, but no meaningful improvement occurred.

**Figure 1:**
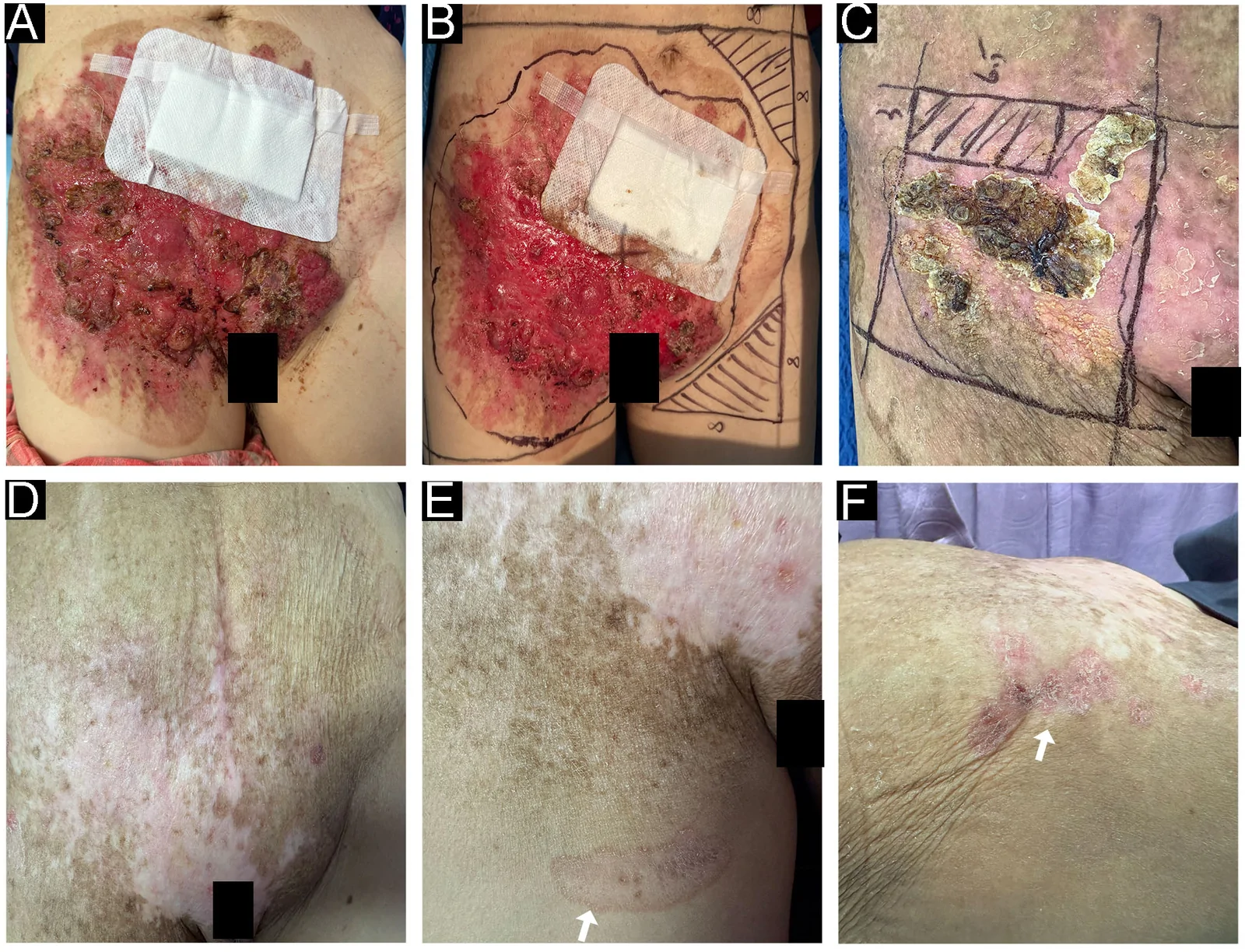
Clinical course and electron beam radiotherapy field design. **(A)** Pretreatment clinical photograph showing a large irregular erythematous and erosive plaque involving the lower abdominal-pelvic skin. **(B)** Skin surface markings for the initial electron field. The marked lines indicate the planned lead-shielding boundary and field-edge design rather than a formally contoured three-dimensional target volume; a 5-mm tissue-equivalent bolus was used to improve surface-dose coverage. **(C)** Marked regression after the initial 43.2-Gy course. The outlined area indicates the second-stage boost field, which was defined according to residual erythema, erosion, crusting, or thickening, with interim MRI used as supporting information. **(D)** Central lower abdominal-pelvic skin within the treated field at nearly 1 year after radiotherapy, showing complete clinical resolution with post-inflammatory pigmentation changes. **(E)** Peripheral lower abdominal-pelvic skin near the initial field edge showing a residual erythematous scaly plaque. **(F)** Adjacent inguinal region showing another residual out-of-field plaque, indicated by the white arrow.

At the initial evaluation, physical examination showed a large, irregular erythematous and erosive plaque involving the lower abdominal wall and adjacent pelvic and inguinal skin. The lesion had ill-defined margins, moist erosion, exudation, crusting, scaling, superficial ulceration, patchy satellite erythema, and heterogeneous red-to-pink discoloration with focal brown crusts. The maximum visible extent was approximately 24 × 21 cm (**Figure 1A**). No additional suspicious lesions were identified in the vulvar, perianal, or umbilical regions; the axillary regions were not specifically examined at that time. The broad curved abdominal-pelvic surface made complete single-field electron coverage technically challenging. The patient had type 2 diabetes mellitus and had recently undergone uncomplicated right cataract surgery. She denied infectious disease, drug allergy, and a family history of malignancy.

### 2.2 Diagnostic assessment

Fungal fluorescence microscopy of skin scrapings was negative. *In vivo* reflectance confocal microscopy showed epidermal spongiosis, focal erosion and architectural disruption, enlarged atypical keratinocyte-like cells suspicious for Paget cells, highly refractive dendritic cells, dilated superficial dermal vessels, and inflammatory cell and melanophage infiltration. These findings supported biopsy rather than continued treatment for simple dermatitis.

**Figure 2:**
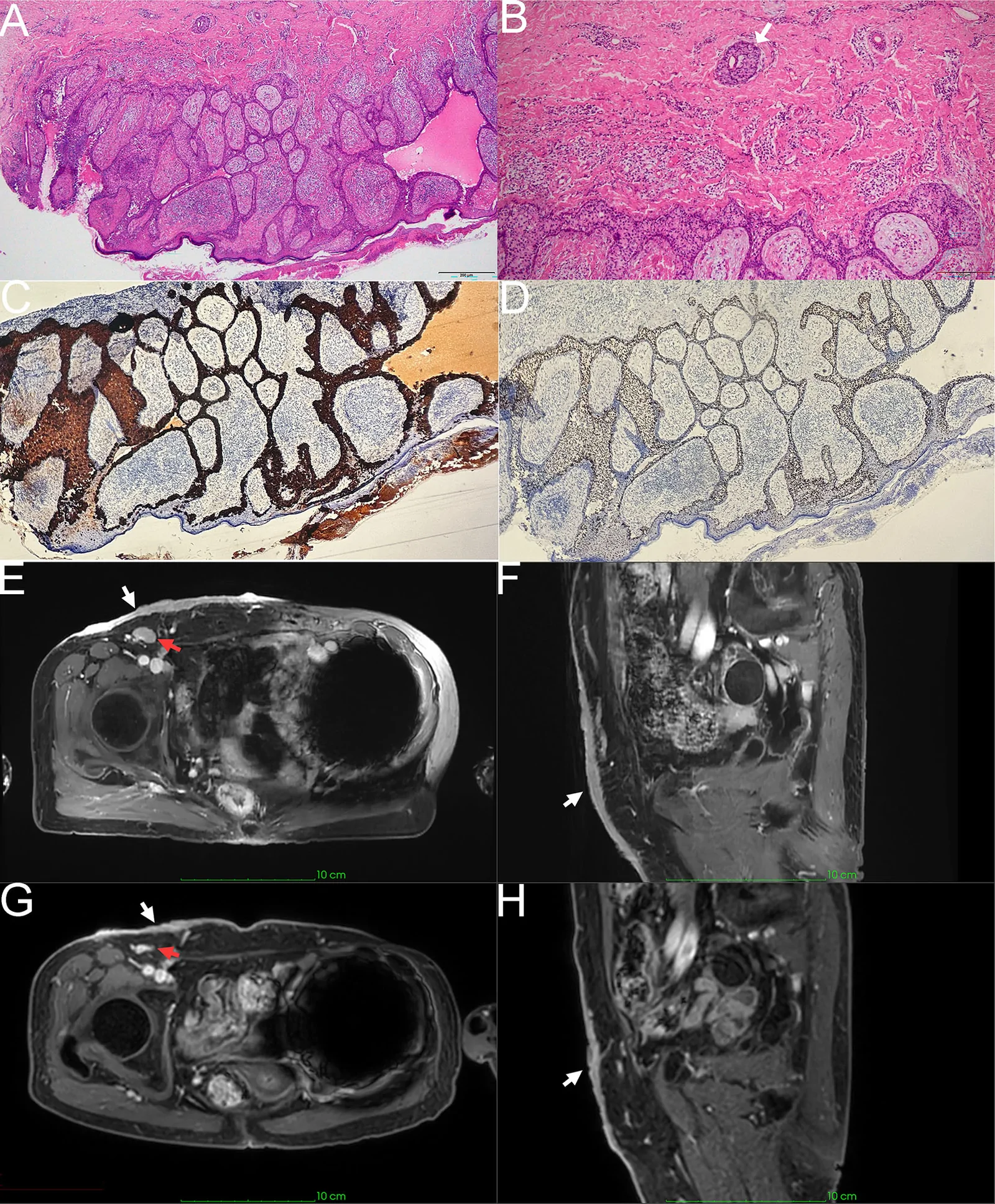
Histopathological, immunohistochemical, and magnetic resonance imaging findings. **(A)** Hematoxylin and eosin staining showing large atypical Paget cells distributed within the epidermis. **(B)** Higher-magnification hematoxylin and eosin staining showing focal extension of atypical cells into the superficial dermis, consistent with microinvasion, indicated by the white arrow. **(C)** Immunohistochemical staining showing CK7 positivity. **(D)** Immunohistochemical staining showing GATA3 positivity. **(E, F)** Pretreatment contrast-enhanced MRI showing irregular thickening and enhancement of the involved abdominal-pelvic wall skin, indicated by white arrows, and enlarged subcutaneous or right inguinal lymph nodes, indicated by red arrows. **(G, H)** Interim contrast-enhanced MRI after the initial 43.2-Gy electron beam radiotherapy course showing regression of the skin lesion and shrinkage of the previously enlarged lymph nodes.

Incisional biopsy revealed hyperkeratosis with parakeratosis and proliferation of large atypical intraepidermal cells with pale cytoplasm, hyperchromatic nuclei, and mitotic activity. Focal extension of atypical cells into the superficial dermis was consistent with microinvasive carcinoma (**Figures 2A, B**). Immunohistochemistry showed positivity for cytokeratin 7 (CK7) and GATA-binding protein 3 (GATA3) (**Figures 2C, D**) and negativity for cytokeratin 20 (CK20) and p40, a marker of squamous differentiation. The Ki-67 proliferation index was approximately 60%, supporting the diagnosis of primary EMPD with focal microinvasion (9–12).

Laboratory tests showed no clinically significant abnormalities in complete blood count, inflammatory markers, liver function, or renal function. Serum carcinoembryonic antigen (CEA), squamous cell carcinoma antigen (SCC antigen), and cytokeratin 19 fragment (CYFRA21-1) were elevated before treatment. Chest computed tomography showed no distant metastasis. Contrast-enhanced magnetic resonance imaging (MRI) showed irregular thickening and enhancement of the involved abdominal-pelvic wall skin and several slightly enlarged enhancing subcutaneous or right inguinal lymph nodes (**Figures 2E, F**). The nodes were considered indeterminate and possibly reactive in the context of the extensive inflamed and erosive skin lesion, as they lacked unequivocal imaging features of metastatic involvement. Given the patient’s advanced age, comorbidities, and the multidisciplinary assessment, ultrasound-guided biopsy was not performed. Neither positron emission tomography-computed tomography (PET-CT) nor dedicated inguinal ultrasonography was obtained before treatment; therefore, nodal metastasis could not be definitively excluded. No underlying visceral malignancy was identified on the available imaging. The final diagnosis was primary ectopic abdominal EMPD with focal microinvasive carcinoma.

### 2.3 Therapeutic intervention

The case was discussed by a multidisciplinary team. Radical surgery was considered unsuitable because of the patient’s advanced age, diabetes mellitus, extensive ill-defined abdominal-pelvic skin involvement, and the anticipated need for wide excision with complex reconstruction. After discussion of surgery, radiotherapy, and other options with the patient and family, individualized electron beam radiotherapy was selected as the primary local treatment, with the aim of symptom relief and durable local control while limiting toxicity.

Radiotherapy was initiated shortly after histopathological confirmation and multidisciplinary evaluation. Treatment was delivered using a Varian linear accelerator. The patient was treated supine and immobilized with an abdominopelvic thermoplastic mask. The treatment field was designed according to the visible lesion, physical examination, standardized clinical photographs, and MRI findings. Skin markings indicated the boundaries of the customized lead shielding and electron field rather than a formally contoured three-dimensional target volume (**Figure 1B**). In the first stage, the main visible lesion and a clinical margin were treated with 12-MeV electrons using a 25 × 25 cm field at a source-to-surface distance of 100 cm. A 5-mm tissue-equivalent bolus was applied to improve surface-dose coverage, and customized 1-cm-thick lead shielding was used to shape the field and protect adjacent normal tissues. A dose of 43.2 Gy in 12 fractions of 3.6 Gy was delivered every other day.

The superficial subcutaneous and right inguinal lymph nodes were not separately delineated as nodal treatment targets but were located within the broad initial field and within the therapeutic depth of the 12-MeV electron beam. They were therefore irradiated, although the dose delivered specifically to the nodes was not separately quantified. After the initial course, the lesion regressed markedly, with reduced moist erythema and erosion and progressive crusting and epithelial healing. Interim contrast-enhanced MRI showed regression of the skin lesion and shrinkage of the previously enlarged lymph nodes (**Figures 2G, H**). Because the nodes were irradiated, their regression could represent either resolution of reactive enlargement or a radiation response of metastatic disease; they therefore remained classified as indeterminate.

The second-stage boost field was defined primarily by direct clinical examination and standardized photographs of the persistent abnormal skin, including residual erythema, erosion, crusting, or thickening, with interim MRI used as supporting information (**Figure 1C**). A further 10 Gy in five consecutive daily fractions of 2 Gy was delivered using 9-MeV electrons, a reduced 10 × 11 cm field, and the same 5-mm bolus approach. The total physical dose was 53.2 Gy.

### 2.4 Follow-up and outcomes

Treatment was well tolerated. The patient developed grade 1 acute radiation dermatitis according to Common Terminology Criteria for Adverse Events version 5.0. No grade 3 or higher skin toxicity, hematologic toxicity, gastrointestinal toxicity, urinary toxicity, or severe treatment-related adverse event occurred.

After completion of radiotherapy, follow-up was based mainly on physical examination and standardized photographic documentation. Approximately one month later, the treated field showed complete clinical resolution, with disappearance of erythema, erosion, exudation, and crusting. Only post-inflammatory hyperpigmentation, mild hypopigmentation, and dry desquamation remained. This complete in-field clinical response has been maintained for nearly one year after radiotherapy, with no clinically evident recurrence within the adequately treated field (**Figure 1D**).

**Figure 3:**
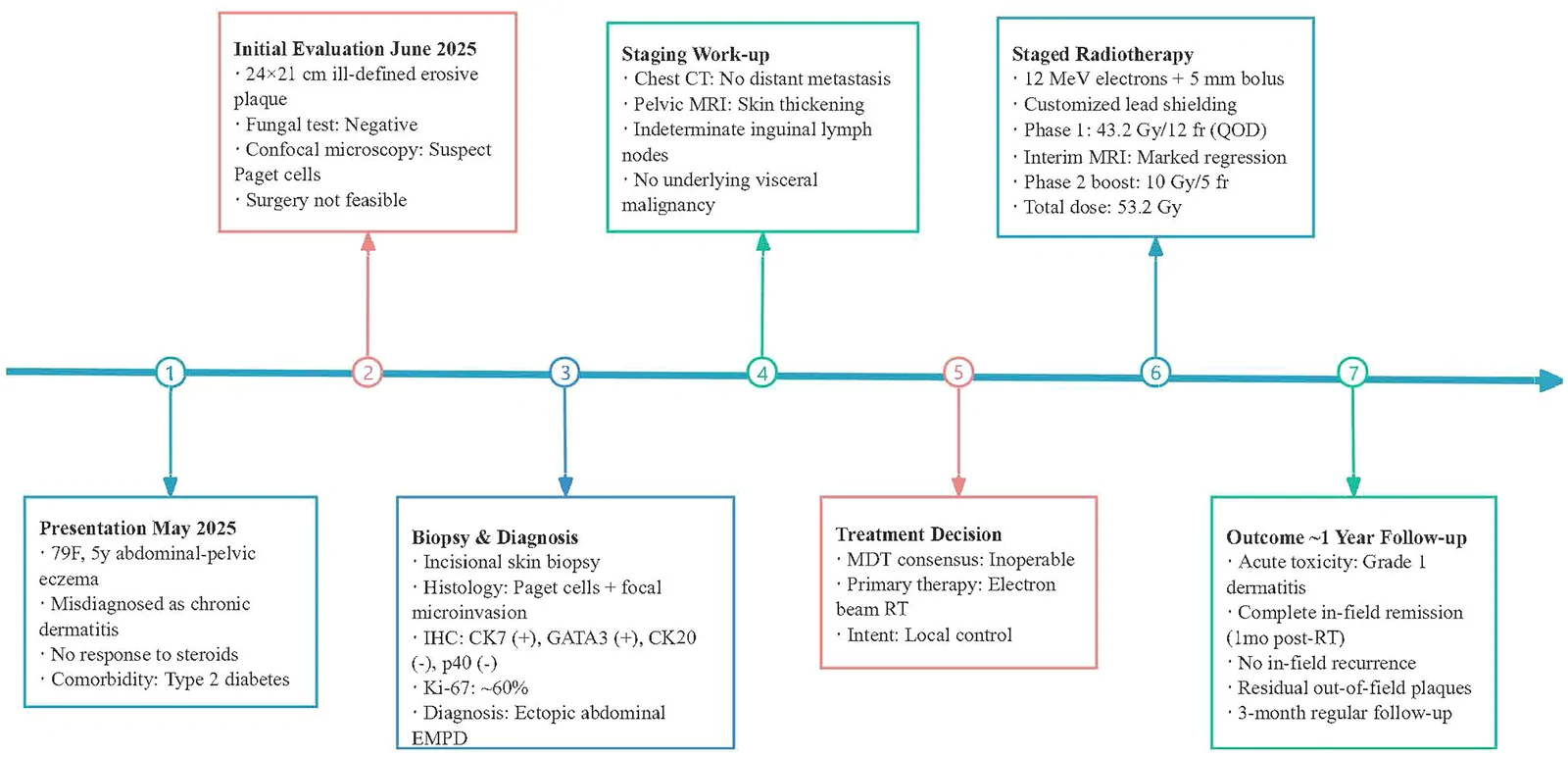
Timeline of the patient’s clinical course. The timeline summarizes the key events from initial presentation and diagnostic evaluation to multidisciplinary treatment decision, staged electron beam radiotherapy, interim response assessment, and approximately 1 year of follow-up. It highlights the response-adapted treatment strategy, in which a localized boost was delivered after marked regression following the initial course, as well as the durable in-field remission and persistent out-of-field plaques observed during follow-up.

Residual erythematous-to-pink scaly plaques persisted outside the initial irradiation field. **Figure 1E** shows a peripheral lower abdominal-pelvic plaque near the field edge, whereas **Figure 1F** shows a residual plaque in the adjacent inguinal region. These lesions were much less extensive than the original disease and lacked extensive moist erosion or exudation, but they suggested possible persistent multifocal or subclinical disease beyond the treated area. The patient remains under clinical and photographic follow-up every three months. Suspected progression would prompt biopsy and restaging. Limited local disease may be considered for surgical excision or additional localized radiotherapy, whereas confirmed nodal or distant progression would lead to consideration of biomarker-guided systemic therapy. Post-treatment CEA, SCC antigen, and CYFRA21–1 values were not reassessed. The overall clinical course is summarized in **Figure 3**.

## 3 Discussion

This case is notable for the unusual location and extensive superficial involvement of ectopic abdominal-pelvic EMPD, as well as the favorable response to response-adapted electron beam radiotherapy in an elderly patient unsuitable for radical surgery. Although the clinically evident disease was centered on the lower abdominal-pelvic skin, EMPD may occur synchronously or metachronously at multiple sites (13). Long-term surveillance should therefore include other apocrine gland-bearing regions, particularly the genital, perianal, axillary, and umbilical areas. Because EMPD may mimic chronic inflammatory dermatoses, persistent or progressive eczema-like lesions that do not respond to standard treatment should undergo early biopsy (14–16).

Ectopic EMPD of the abdominal or umbilical skin is rare and clinically heterogeneous. A reported patient with invasive umbilical EMPD subsequently developed nodal, pulmonary, and osseous metastases and achieved a complete response to pembrolizumab after a high tumor mutational burden was identified (17). In contrast, our patient had a much larger but predominantly superficial lower abdominal-pelvic lesion with only focal microinvasion and no confirmed distant disease. These contrasting presentations indicate that management should be individualized according to disease extent, invasion depth, nodal or distant involvement, surgical feasibility, and molecular characteristics.

Surgical resection is generally preferred for localized EMPD, but recurrence can occur because Paget cells may spread multifocally beyond clinically visible margins (18, 19). Sentinel lymph node biopsy or targeted nodal assessment may be considered in selected invasive or clinically node-suspicious disease (5, 20). In the present patient, extensive skin involvement, diabetes, advanced age, ill-defined borders, and expected reconstructive morbidity made radical surgery unattractive. The absence of pathological nodal confirmation limited accurate staging and remains an important uncertainty.

Previous studies have reported favorable local control with definitive radiotherapy for EMPD, particularly for *in situ* or superficially invasive disease, although the evidence is based mainly on small retrospective series and case reports (6, 21–25). The favorable response in this patient was likely related to the predominantly intraepidermal and superficially microinvasive disease, the use of electron beams appropriate for a superficial target, the 5-mm bolus used to improve surface dose, and a response-adapted boost to persistent visible disease. The two-stage strategy allowed the broad symptomatic lesion to be treated first, followed by a smaller boost field after clinical and MRI response assessment.

The principal clinical insight is that adequate surface dose within the treated field does not ensure adequate geographic coverage of peripheral or clinically occult disease. Electron beams are well suited for superficial cutaneous tumors because they provide high surface and shallow tissue dose with relative sparing of deeper organs. However, extensive EMPD on a curved abdominal-pelvic surface creates geometric uncertainty. Oblique incidence, imperfect bolus contact, shielding, setup reproducibility, and field-edge effects may compromise peripheral coverage. In this case, complete and durable remission within the irradiated field contrasted with persistent plaques outside the field, indicating that bolus use cannot compensate for incomplete mapping, inadequate margins, or geographic miss.

For similar cases, pretreatment mapping should be strengthened using careful dermatologic examination, standardized photographs, dermoscopy or reflectance confocal microscopy, MRI, and peripheral mapping biopsies when feasible. Field design should consider sufficiently comprehensive margins, reproducible immobilization, customized shielding, bolus conformity, and matched-field planning when more than one electron field is required. Surface mold brachytherapy, three-dimensional printed bolus or applicators, surface-guided setup, and modern conformal techniques may improve coverage for extensive or irregular lesions (26–29).

If a limited, discrete, biopsy-confirmed residual or recurrent lesion becomes technically resectable, surgical excision could be reconsidered. Surgery has not been pursued for the current residual plaques because they remain ill-defined and potentially multifocal, while the patient’s age, diabetes, and anticipated wound-healing and reconstructive risks remain relevant. Additional localized radiotherapy may be considered for superficial unresectable progression after improved mapping and treatment planning. Confirmed nodal or distant progression would prompt molecular characterization and multidisciplinary consideration of systemic treatment, including chemotherapy, human epidermal growth factor receptor 2 (HER2)-directed therapy for HER2-positive disease, or immune checkpoint inhibition in an appropriate biomarker-defined context (30–32).

This case has limitations. It is a single case with limited follow-up, no post-boost imaging, no pathological confirmation of the residual out-of-field plaques, and no nodal biopsy, dedicated inguinal ultrasonography, or PET-CT staging. Post-treatment tumor-marker values were unavailable. The axillary regions were not specifically examined at the initial evaluation. Complete surface-dose and field-edge dosimetric data and systematic peripheral mapping biopsies were also unavailable. Despite these limitations, the case demonstrates that response-adapted electron beam radiotherapy can achieve meaningful in-field control while illustrating the consequences of incomplete geographic coverage.

## 4 Conclusion

Response-adapted two-stage electron beam radiotherapy produced complete clinical resolution within the treated field in an elderly patient with inoperable primary ectopic abdominal EMPD with focal microinvasion. Persistent out-of-field plaques emphasize that successful radiotherapy for extensive curved superficial lesions requires not only adequate surface dose but also accurate pretreatment mapping, comprehensive field coverage, bolus conformity, reproducible shielding, and careful field-edge planning.

## 5 Patient perspective

The patient reported that persistent erosion, exudation, and pruritus had impaired daily comfort and hygiene for several years. She considered radiotherapy tolerable and appreciated the non-invasive nature of treatment. After the treated lesion resolved, she reported substantial symptomatic relief and improved quality of life. She understood that residual scaly plaques outside the original field required continued follow-up and possible further treatment, but expressed overall satisfaction with the in-field response.

## Data Availability

The raw data supporting the conclusions of this article will be made available by the authors, without undue reservation.
